# Parenteral Nutrition Management from the Clinical Pharmacy Perspective: Insights and Recommendations from the Saudi Society of Clinical Pharmacy

**DOI:** 10.3390/pharmacy14010016

**Published:** 2026-01-26

**Authors:** Nora Albanyan, Dana Altannir, Osama Tabbara, Abdullah M. Alrajhi, Ahmed Aldemerdash, Razan Orfali, Ahmed Aljedai

**Affiliations:** 1 Clinical Pharmacy Department, King Fahad Medical City, Riyadh 12231, Saudi Arabia; nalbanyan@gmail.com; 2 Research Center Department, King Fahad Medical City, Riyadh 12231, Saudi Arabia; 3 College of Medicine, Alfaisal University, Riyadh 11533, Saudi Arabia; 4 IVPN Network, Abu Dhabi, United Arab Emirates; 5 Department of Pharmacy Practice, College of Pharmacy, Alfaisal University, Riyadh 11533, Saudi Arabia; 6 Department of Pharmacy, Oncoclinic, Riyadh 01309, Saudi Arabia; 7 Department of Clinical Pharmacy, College of Pharmacy, King Saud University, Riyadh 11451, Saudi Arabia; 8 Department of Pharmacology, College of Medicine, Imam Mohammad Ibn Saud Islamic University, Riyadh 13317, Saudi Arabia

**Keywords:** parenteral nutrition, clinical pharmacy, Saudi healthcare, TPN, clinical pharmacist ratio, health management system

## Abstract

Parenteral nutrition (PN) is essential for patients who are unable to tolerate oral or enteral feeding, providing them with necessary nutrients intravenously, including dextrose, amino acids, electrolytes, vitamins, trace elements, and lipid emulsions. Clinical pharmacists (CPs) play a critical role in PN management by ensuring proper formulation, monitoring therapy, preventing complications, and optimizing patient outcomes. In Saudi Arabia, limited literature exists on CPs’ involvement in total parenteral nutrition (TPN) administration, health information management (HIM) systems, and pharmacist staffing ratios. This paper examines the evolving role of CPs in PN management, addressing key challenges such as the optimal patient-to-CP ratio, the impact of HIM systems on PN prescribing, and the advantages and limitations of centralized versus decentralized PN prescription models. It highlights the need for standardized staffing levels, structured pharmacist training, and improved HIM integration to enhance workflow efficiency and prescribing accuracy. Additionally, the study examines how the adoption of advanced HIM systems can streamline documentation, reduce prescribing errors, and enhance interdisciplinary collaboration. This paper provides a framework for optimizing PN delivery, enhancing healthcare quality, and strengthening CPs’ contributions to nutrition support by addressing these factors. Implementing these recommendations will improve patient outcomes and establish a more efficient PN management system in Saudi Arabia, reinforcing the vital role of CPs in multidisciplinary care.

## 1. Introduction

Parenteral nutrition (PN) therapy is the adequate intravenous delivery of nutritious mixtures containing water, dextrose, amino acids, lipid emulsions (fat), electrolytes, vitamins, and trace elements to patients who cannot tolerate oral or enteral feeding [1,2]. PN includes both total parenteral nutrition (TPN), typically delivered via central venous access and intended to meet full daily nutritional needs, and peripheral parenteral nutrition (PPN), delivered via peripheral access and generally used for short-term or supplemental support [1,2]. PN is essential for patients with various medical conditions, such as premature neonates, critically ill individuals, and those with slight gastrointestinal function loss [3,4]. Ideally, a multidisciplinary team known as the Nutrition Support Team (NST), comprising, but not limited to, a physician, pharmacist, dietitian, and nurse, provides PN therapy to optimize patient care [1,3].

Clinical Pharmacists (CPs) can play a key role in the NST by significantly contributing to the care of patients receiving PN therapy [5,6]. They possess specialized knowledge about the physicochemical compatibilities of parenteral solutions, compounding standards, pharmacotherapy principles, and pharmaceutical care practices [7]. Pharmacists can further develop their expertise by undergoing specialized training in nutritional support, known as Nutrition Support Pharmacy (NSP) [3]. NSP is a specialty that optimizes nutrition support therapy outcomes [3]. The role of CPs in PN therapy can vary across healthcare settings, depending on their position, education, and practice environment [3]. This role can range from limited to compounding PN formulations to providing direct patient care [3].

Due to physicians’ limited involvement in PN, clinical pharmacists have taken on a more active role in managing PN patients over the past four decades. This pharmacist’s involvement is essential to optimize patient outcomes, minimize complications, and enhance healthcare delivery [1]. The COVID-19 pandemic further highlighted the critical need for resilience in healthcare services, including PN management. Centralized service models faced significant challenges due to frequent absenteeism, threatening the continuity of care. These disruptions underscored the importance of comprehensive training and competency programs for all clinical pharmacists in managing and prescribing PN. By ensuring that all pharmacists are proficient in these areas, healthcare systems can enhance service continuity and resilience, mitigating risks associated with absenteeism and ensuring the reliable delivery of healthcare services.

Given the limited literature in Saudi Arabia on PN service models, health information management (HIM) support for PN prescribing, and pharmacist staffing considerations, this paper summarizes insights and recommendations from the Saudi Society of Clinical Pharmacy. This paper discusses global PN practices within Saudi healthcare regulations, workforce models, and digital health infrastructure, thereby providing locally actionable guidance rather than a purely descriptive synthesis. We focus on three practical domains: (i) aligning pharmacist staffing with PN workload, (ii) leveraging HIM systems to improve prescribing accuracy and workflow, and (iii) evaluating centralized versus decentralized PN prescribing models. These recommendations aim to support safer, more efficient PN delivery and strengthen clinical pharmacists’ contributions within multidisciplinary nutrition support services in Saudi Arabia.

Together, pharmacist workforce capacity, health information management systems, and PN delivery models represent interdependent components of safe and effective PN services, and are therefore discussed sequentially to illustrate their combined impact on clinical outcomes.

### Patient-to-Clinical Pharmacist Ratio

Studies have shown that pharmacist monitoring of patients on PN therapy led to better clinical responses to PN and decreased PN-related costs [8,9].

The role of a nutritional support pharmacist is quite intensive, including monitoring patients receiving nutritional support through reports or patient visits in collaboration with other healthcare professionals. This role involves establishing appropriate monitoring parameters in line with the patient’s nutrition care plan to optimize therapy and achieve the best outcomes.

Additionally, the nutritional support pharmacist is responsible for regularly reassessing the suitability of the nutritional support therapy, including the feeding formulation, route of delivery, and method of delivery. They must evaluate its efficacy and safety using relevant monitoring parameters and thoroughly review concurrent medications to prevent significant drug-induced metabolic disorders.

In addition, monitoring vitamin and trace element levels in patients requiring specialized nutrition support is crucial, highlighting pharmacists’ unique skills through additional nutrition support training [8].

Clinical Pharmacists (CPs) contribute substantial value to the healthcare team, reducing hospital mortality, drug costs, length of stay, and adverse drug reactions [10]. Despite growing recognition of the value that CPs add to primary and ambulatory care practices, a standardized staffing model has yet to be established [10], particularly for clinical pharmacists and PN prescriptions. For instance, the recent American College of Clinical Pharmacy (ACCP) research aimed at identifying pharmacist-to-patient ratios for the successful provision of clinical pharmacy services did not address the ideal Nutrition Support Pharmacist (NSP)-to-patient ratio due to the lack of literature [11].

Given the complexity and critical nature of PN therapy, especially for vulnerable populations like neonates, it is essential to establish clear guidelines for staffing ratios and pharmacist qualifications. The following recommendations are proposed to enhance the effectiveness of PN services based on existing literature and experts’ opinions:

- Identify Optimal Patient-to-NSP Ratios: Conduct targeted studies to determine the ideal patient-to-clinical pharmacist ratio for safe and effective PN delivery. This should consider factors such as hospital capacity, patient demographics (e.g., a high proportion of neonatal patients), and the complexity of cases.
- Set Qualifications for PN Services: Define the necessary qualifications for pharmacists involved in PN services, such as requiring a residency in nutritional support pharmacy or equivalent specialized training, to ensure that they can manage PN therapy competently.
- Tailor Staffing Models to Hospital Capacity: Consider the size and capacity of the hospital when determining the need for dedicated PN Clinical Pharmacists. For example, hospitals with 100–499 beds may require different staffing models than larger hospitals with 500–999 beds.
- Create Multidisciplinary Teams: Establish multidisciplinary teams in hospitals, with a clinical NSP leading the PN approach, to ensure comprehensive management of PN therapy.
- Divide Workload Appropriately: Allocate tasks based on the complexity of prescriptions, the training of pharmacists, and the hospital’s size to prevent burnout and optimize patient care.
- Conduct Further Research: More research is needed to establish evidence-based NSP-to-patient ratios and ensure resource allocation aligns with patient needs, particularly in settings with high neonatal PN patients.

Different hospital specialties have varying demands for PN therapy. Oncology patients often require complex and individualized PN regimens, which can increase the time and effort needed from clinical pharmacists. Similarly, intensive care units (ICUs) may present more challenging cases due to the critical nature of patients’ conditions [11]. Understanding how these factors influence the patient-to-clinical pharmacist ratio is essential for developing appropriate staffing models.

For instance, according to a large-scale study published by the American College of Clinical Pharmacy, the full-time clinical pharmacist-to-patient ratio is maintained at 20 patients per pharmacist in the oncology and Intensive Care units [11]. In contrast, this ratio is adjusted for stable patients to 30 patients per full-time clinical pharmacist [11]. Overall, it is reasonable for each full-time clinical pharmacy nutritionist to manage a mix of 25–30 patients requiring parenteral nutrition, depending on the level of care required. This variability highlights the need to tailor the pharmacist-to-patient ratio according to the complexity and intensity of the patient’s conditions, ensuring optimal care and resource allocation.

## 2. Health Information Management Systems

There are two types of health information management systems. The first is the stand-alone system, which is used only for e-prescribing and is not connected to the patient’s electronic medical record [12]. The other is the integrated systems, part of a comprehensive electronic health record system [12]. Integrated HIM systems combine all patient information and clinical tools in a single platform and can streamline the prescribing process and reduce errors [12]. Conversely, stand-alone systems may require additional data entry and coordination time among healthcare providers [12]. Pharmacy leaders should prioritize developing and implementing integrated PN systems to prevent transcription errors that can occur with stand-alone systems.

At Johns Hopkins All Children’s Hospital, 22% of PN orders needed clarification due to errors, and pharmacists spent an average of 10 min per order correcting these errors [13]. Standardizing PN and moving to electronic ordering significantly decreased ordering errors and processing time [13]. This transition also greatly improved resource efficiency by reducing the frequency of blood draws, making the transition more cost-efficient [13].

A study conducted in Saudi Arabia identified three significant barriers to implementing hospital information systems, including financial, organizational, and regulatory challenges. Financial challenges were found to be the most significant, due to high implementation and operational management costs, as well as delays in the implementation timeline. This is particularly concerning among governmental hospitals due to decreased funding. Organizational barriers, such as a lack of a proper HIM adoption strategy and inadequate staff training, may impede the implementation of HIM and decrease the program’s efficiency if not addressed. Finally, regulatory barriers include the lack of governmental laws and policies that target the mandatory implementation of HIM in hospitals [14].

Recommendations:

- Investigate system impacts: Study the effects of stand-alone and integrated HIM systems on PN standardization, focusing on ordering errors and processing time as key performance indicators.
- Implement integrated systems: Promote the use of integrated HIM systems to streamline PN prescribing, reduce errors, and improve workflow efficiency.
- Conduct comparative studies: Compare clinical outcomes and resource efficiency between hospitals using stand-alone versus integrated systems.
- Develop standardized protocols: Create and implement standardized protocols and training programs for HIM systems in PN prescribing to ensure consistent practice and patient safety.

## 3. Model of Parenteral Nutrition Prescription (Centralized vs. Decentralized)

The specialization and expertise of clinical pharmacists differ significantly between centralized and decentralized models. In a centralized approach, only a specialized team of clinical pharmacists with extensive training and experience in PN prescribing handles all PN orders. In contrast, a decentralized model allows any clinical pharmacist trained in PN prescribing to manage orders, although they may not have the same level of specialization [15]. This impacts consistency and quality, with centralized systems typically offering higher consistency and potentially higher quality of PN orders due to specialized knowledge and focus. Decentralized systems might experience variability in order quality as different pharmacists with varying expertise and experience may handle the orders [15,16]. Efficiency and turnaround time also vary; centralized systems often have faster processing times due to the expertise of a dedicated team and streamlined workflows, while decentralized systems may experience fluctuating processing times based on the availability and workload of individual pharmacists [15].

Resource utilization is another divergence; centralized models concentrate resources, potentially leading to more efficient use of specialized skills, whereas decentralized models distribute the workload among pharmacists, balancing the overall workload but possibly not utilizing specialized skills as efficiently [15]. Training and education are more straightforward to maintain at high levels within a smaller, focused team in centralized models. Still, decentralized models require broader training programs to ensure all pharmacists are adequately trained in PN prescribing, making it more resource-intensive [1,15]. Flexibility and coverage also differ; centralized models offer less flexibility as the specialized team may not always be available, while decentralized models provide greater flexibility and coverage by training more pharmacists to handle PN prescribing, reducing dependence on a specific team [15].

Continuity of care is another consideration; centralized systems provide better continuity of care for patients receiving PN as the same specialized team manages their nutrition, whereas decentralized systems may face fragmented care if different pharmacists handle PN orders at different times [15,17]. Communication and coordination are easier to maintain within a smaller, specialized team in centralized models. Still, decentralized models require robust communication systems to ensure consistency and coordination among all pharmacists involved in PN prescribing [15]. Finally, accountability differs; centralized models have clear accountability within a specialized team for PN-related outcomes, while decentralized models have shared accountability among all trained pharmacists, potentially diluting individual responsibility [15].

A crucial point is that decentralized care is more cost-effective than centralized care where a Canadian hospital case study showed an annual saving of $11,911 (USD) when implementing the decentralized model [15]. This is a crucial challenge for implementing centralized care models in hospitals, especially non-governmental hospitals. Future research should explore the long-term impact of pharmacist-led PN management on patient outcomes and healthcare costs in Saudi Arabia.

Recommendations:

1. Assess daily PN orders: The decision to adopt a centralized or decentralized model should be based on the total number of daily parenteral nutrition (PN) orders and the type of PN service provided. A centralized method is recommended if PN orders exceed 20 per day or home PN patients are being managed. Conversely, a decentralized method may be more practical and efficient if PN orders are fewer than 20 per day and patients require short-term PN. A dedicated staff member is also needed to manage these orders if home PN services are provided.
2. Observe long-term outcomes: Monitor patient outcomes over time to assess the effectiveness of different PN prescribing models.
3. Develop guidelines: Create best practice guidelines for implementing centralized and decentralized PN prescribing models, ensuring flexibility and adaptability to different hospital sizes and patient needs.
4. Ensure continuous education: Provide ongoing training and education for pharmacists in centralized and decentralized models to maintain high standards of care.

Table 1 summarizes the key differences between centralized and decentralized PN prescribing models discussed in Section 3. In general, centralized PN models are preferable for high-risk patients and complex formulations, whereas decentralized models may be appropriate for stable patients with standardized regimens, provided adequate clinical pharmacist oversight is available.

## 4. Conclusions

In conclusion, optimizing parenteral nutrition (PN) therapy is essential for enhancing patient outcomes and improving healthcare delivery. By investigating the patient-to-clinical pharmacist ratio, the type of health information management system, and the model of PN prescribing (centralized vs. decentralized), healthcare institutions can develop strategies to enhance the efficacy of PN prescriptions by pharmacists. Implementing the recommended practices, such as determining optimal NSP-to-patient ratios, utilizing integrated HIM systems, and choosing the appropriate PN prescribing model based on daily order volumes, can lead to more efficient and effective PN therapy. Continuous education and adherence to best practice guidelines are essential to maintaining high standards of care and ensuring the success of PN therapy across various healthcare settings.

## Figures and Tables

**Table 1:** Centralized compared to decentralized models.

Aspect	Centralized Model	Decentralized Model
Expertise	Handled by a specialized team of highly trained pharmacists, ensuring high consistency and quality in PN orders.	It is managed by any trained pharmacist, which may result in variability in order quality due to differing expertise.
Efficiency	Faster processing due to streamlined workflows and expertise.	Processing times fluctuate based on individual pharmacist availability and workload.
Resource Utilization	Concentrates resources and optimizes the use of specialized skills.	Distributes workload across pharmacists, potentially less efficient use of specialized expertise.
Training	Easier to maintain high-level training within a focused team.	Requires broader training programs, making it more resource-intensive.
Flexibility	Less flexible as it depends on the availability of a specialized team.	More flexible, with broader coverage by training more pharmacists in PN prescribing.
Continuity of Care	It offers better continuity, as the same specialized team manages patient nutrition.	May face fragmented care if different pharmacists handle orders at different times.
Communication	Easier coordination within a smaller, specialized team.	Requires robust communication systems to ensure consistency among all pharmacists involved.
Accountability	Clear accountability lies with the specialized team.	Shared accountability among multiple pharmacists, potentially diluting individual responsibility.
Cost	More expensive to implement and sustain.	More cost-effective, with studies showing significant annual savings (e.g., $11,911 in a Canadian hospital).

## Data Availability

No new data were created or analyzed in this study.
